# Pembrolizumab versus bevacizumab plus modified FOLFOX6 in metastatic MSI-H/dMMR colorectal cancer: a multicenter retrospective study with CT evaluation

**DOI:** 10.3389/fonc.2025.1570457

**Published:** 2025-04-04

**Authors:** Jiaqi Chen, Weiguang Yu, Xiaobo Xia, Yang Zhao, Qiang Tang, Yunxiang Zhang, Yijie Zhang, Haoyu Zhang, Zhong Zhang, Xiaoyan Zhang, Jianghua Lou

**Affiliations:** ^1^ Department of Medical Image, Henan Provincial People’s Hospital, Zhengzhou, China; ^2^ Department of Emergency Surgery and Orthopaedics, The First Affiliated Hospital, Sun Yat-sen University, Guangzhou, China; ^3^ Department of Gastrointestinal Surgery, Henan Provincial People’s Hospital, Zhengzhou, China; ^4^ Department of Cancer Center, Henan Provincial People’s Hospital, Zhengzhou, China; ^5^ Department of Hepatobiliary Surgery, Henan Provincial People’s Hospital, Zhengzhou, China

**Keywords:** pembrolizumab, bevacizumab, colorectal cancer, FOLFOX6, overall survival

## Abstract

**Objective:**

The optimal therapeutic strategy for metastatic microsatellite instability-high/mismatch repair-deficient (MSI-H/dMMR) colorectal cancer (CRC) remains uncertain. This multicenter retrospective study compared the efficacy and safety of pembrolizumab monotherapy versus bevacizumab combined with modified FOLFOX6 (mFOLFOX6) in this molecularly defined population.

**Methods:**

Consecutive patients with metastatic MSI-H/dMMR CRC treated with pembrolizumab or bevacizumab plus mFOLFOX6 at two tertiary centers (2017–2024) were analyzed. Dual primary endpoints included overall survival (OS) and progression-free survival (PFS); secondary endpoints encompassed incidence of grade ≥3 treatment-emergent adverse events (AEs).

**Results:**

Among 58 eligible patients (PE: n=30; BF: n=28), the PE cohort demonstrated a significantly higher objective response rate (ORR) compared to the BF cohort (XX% vs XX%, p=0.030) after a median follow-up of 18.0 months (IQR: 1.0–24.0). Survival analyses revealed superior outcomes in the PE cohort, with a median OS of 12.0 months (95% CI: 10.2–14.1) versus 8.8 months (95% CI: 7.1–9.6) in the BF cohort (HR=0.55, 95% CI: 0.29–0.56; p=0.02). Similarly, median PFS was prolonged in the PE cohort (7.0 months, 95% CI: 5.3–9.3) relative to the BF cohort (3.7 months, 95% CI: 2.2–5.4; HR=0.46, 95% CI: 0.24–0.89; p<0.001). No statistically significant intergroup differences were observed in grade ≥3 treatment-emergent AE rates.

**Conclusion:**

Pembrolizumab monotherapy significantly improved survival over bevacizumab-based chemotherapy in metastatic MSI-H/dMMR CRC, with a manageable safety profile. These results reinforce PD-1 inhibitors as first-line therapy for this population, while highlighting tumor mutation burden (TMB) and tumor burden as critical biomarkers for personalized strategies.

## Introduction

Microsatellite instability-high/mismatch repair-deficient (MSI-H/dMMR) colorectal cancer (CRC) accounts for approximately 5% of metastatic CRC cases and arises from single-nucleotide mismatches or functional impairment of DNA mismatch repair mechanisms (1–3). Accumulating evidence (4, 5) highlights the distinct clinicopathological features of MSI-H/dMMR CRC, including its predilection for right-sided colonic origin, low prevalence, and intrinsic resistance to conventional chemotherapy. Despite emerging level 1 evidence supporting immune checkpoint inhibitors (ICIs) as first-line therapy for MSI-H/dMMR CRC, chemotherapy remains a widely utilized conventional approach (6, 7), underscoring persistent challenges in optimizing therapeutic strategies for metastatic disease (6, 8, 9). Notably, MSI-H/dMMR tumors exhibit heightened immunogenicity due to neoantigen accumulation, rendering them particularly responsive to PD-1/PD-L1 blockade (10, 11). Mechanistically, PD-1 inhibitors disrupt the interaction between PD-1 on cytotoxic T cells and PD-L1 on tumor cells, thereby restoring antitumor immune activity (1, 2, 11). The phase II KEYNOTE-164 trial (12) demonstrated durable clinical benefits of pembrolizumab in pretreated metastatic MSI-H/dMMR CRC, with an objective response rate (ORR) of 33% (95% CI: 21–46), median progression-free survival (PFS) of 2.3 months (95% CI: 2.1–8.1), and median overall survival (OS) of 31.4 months (95% CI: 21.4–not reached) after 31.3 months of follow-up. Subsequent phase III trial (13) further established pembrolizumab’s superiority over chemotherapy in treatment-naïve patients, reporting a median PFS of 16.5 months (95% CI: 5.4–32.4) versus 8.2 months (95% CI: 6.1–10.2; HR=0.60, p=0.0002), alongside a favorable safety profile. Conversely, bevacizumab—a humanized anti-VEGF monoclonal antibody—combined with chemotherapy has shown modest efficacy in this population, though its role remains contentious (12, 14).

Despite these advances, critical gaps persist. While pembrolizumab monotherapy has demonstrated robust antitumor activity, the clinical utility of bevacizumab combined with modified FOLFOX6 (mFOLFOX6) in MSI-H/dMMR CRC remains underexplored, with limited comparative data on survival outcomes and toxicity profiles (12, 13). Specifically, it remains unclear whether pembrolizumab confers superior survival benefits over bevacizumab-based regimens in this molecularly defined subset. To address this uncertainty, we conducted a multicenter retrospective study evaluating the efficacy and safety of pembrolizumab versus bevacizumab plus mFOLFOX6 in patients with metastatic MSI-H/dMMR CRC.

## Materials and methods

### Patient eligibility

Retrospective clinical data were extracted from two affiliated medical institutions for patients diagnosed with advanced microsatellite instability–high/mismatch repair-deficient (MSI-H/dMMR) colorectal adenocarcinoma between January 2017 and August 2024. The study cohort comprised consecutive patients treated with either pembrolizumab monotherapy (PE cohort) or bevacizumab combined with modified FOLFOX6 chemotherapy (BF cohort). Participants were required to meet the following inclusion criteria: histologically or cytologically confirmed metastatic colorectal adenocarcinoma with MSI-H/dMMR status; radiologically measurable disease per modified Response Evaluation Criteria for Solid Tumors (mRECIST v1.1); adequate organ function (cardiopulmonary, hepatic, and renal), defined as: left ventricular ejection fraction ≥50%; serum creatinine ≤1.5× upper limit of normal; total bilirubin ≤1.5× upper limit of normal. Eastern Cooperative Oncology Group performance status (ECOG PS) of 0 or 1. Patients were excluded based on: insufficient baseline clinical documentation; history of other active malignancies within 5 years; prior systemic therapy with monoclonal antibodies, anti-PD-1/PD-L1/PD-L2 agents, or multiagent chemotherapy regimens; active autoimmune disorders requiring immunosuppressive therapy (e.g., rheumatoid arthritis, systemic lupus erythematosus); clinically significant comorbidities, including: uncontrolled diabetes mellitus (HbA1c >9%), obesity (body mass index [BMI] ≥35 kg/m²), uncontrolled coagulopathy necessitating therapeutic anticoagulation, symptomatic interstitial lung disease, or New York Heart Association class III/IV cardiac dysfunction; acute intestinal obstruction (≤12 months prior to enrollment); concurrent severe infections (e.g., systemic inflammatory response syndrome, active pulmonary tuberculosis); protocol nonadherence (treatment discontinuation unrelated to disease progression or toxicity, loss to follow-up); documented psychiatric or cognitive impairment affecting treatment compliance.

### Study design and management

This multicenter retrospective cohort study evaluated patients with metastatic MSI-H/dMMR CRC who received either pembrolizumab monotherapy (PE cohort) or bevacizumab combined with modified FOLFOX6 chemotherapy (BF cohort). Treatment protocols were structured as follows: pembrolizumab regimen (PE): Patients received intravenous pembrolizumab at a dose of 500 mg/m^2^ administered over 1 hour every 2 weeks (q2w), consistent with the dosing schedule outlined in the KEYNOTE-164 trial (13); bevacizumab plus modified FOLFOX6 (BF): Patients were administered 5 mg/kg intravenous bevacizumab over 30 minutes (q2w) (15), followed by the modified FOLFOX6 regimen comprising 85 mg/m² oxaliplatin, 200 mg/m² leucovorin infused over 2 hours, and 2400 mg/m² fluorouracil delivered via continuous 48-hour infusion, repeated every 2 weeks as per established protocols, as described by Venook et al. (16) and Cremolini et al. (15)). Treatment continuation was contingent upon disease progression, intolerable toxicity, or death, with no predefined maximum duration. Routine clinical management, including dose modifications and supportive care, adhered to institutional guidelines under the supervision of the treating oncology team.

### Outcomes and evaluations

The primary endpoints of this study included overall survival (OS) and progression-free survival (PFS). OS was defined as the duration from the initiation of treatment until the time of death from any cause, while PFS was measured from the first dose until the occurrence of disease progression or death, whichever occurred first. Disease progression and treatment response were assessed using contrast-enhanced CT scans, with tumor measurements performed at baseline and every 8 weeks (±1 week) thereafter, according to mRECIST v1.1 criteria.

Following the initial treatment dose, survival data were collected and monitored at intervals of four weeks. Safety profiles were continuously evaluated using the Common Terminology Criteria for Adverse Events (CTCAE version 4.0), with assessments conducted at least every two weeks during the first 24 weeks of treatment, and subsequently every eight weeks until the final follow-up or the patient’s death. Tumor mutation status was assessed through both immunohistochemistry and polymerase chain reaction (PCR) methods. The dMMR was established based on the loss of expression of at least one mismatch repair protein, as determined via immunohistochemistry (17). The identification of MSI-H was conducted using PCR, following previously reported methodologies (18). The expression levels of tumor PD-L1 were quantified using the combined positive score (CPS) method, consistent with established protocols in the literature (19). Tumor mutation burden (TMB) was also measured in this study. We performed a *post hoc* analysis using available next-generation sequencing (NGS) data for a subset of patients. Patients were stratified into high TMB (≥10 mutations/megabase) and low TMB (<10 mutations/megabase) subgroups based on established thresholds for MSI-H/dMMR CRC (20, 21).

### Statistical analysis

Descriptive statistics summarized baseline characteristics and toxicities, with categorical variables expressed as frequencies (%) and continuous variables as medians (IQR) or means (SD). Median follow-up was calculated using the reverse Kaplan-Meier method. Survival outcomes (OS, PFS) were analyzed via Kaplan-Meier curves and log-rank tests. Cox proportional hazards models estimated hazard ratios (HR) with 95% CIs, adjusting for age, sex, BMI, ECOG PS, tumor location, PD-L1 expression (CPS ≥1 vs. <1), and comorbidities (e.g., uncontrolled diabetes). Continuous variables were modeled linearly unless nonlinearity was detected. Proportional hazards assumptions were validated using Schoenfeld residuals (all p > 0.05). Treatment was analyzed as a time-dependent variable. Sensitivity analyses excluded patients with missing data (n=3). Two-sided p-values <0.05 were considered significant. Analyses used SAS 9.4 and R 4.4.2.

## Results

### Demographic characteristics

**Figure 1:**
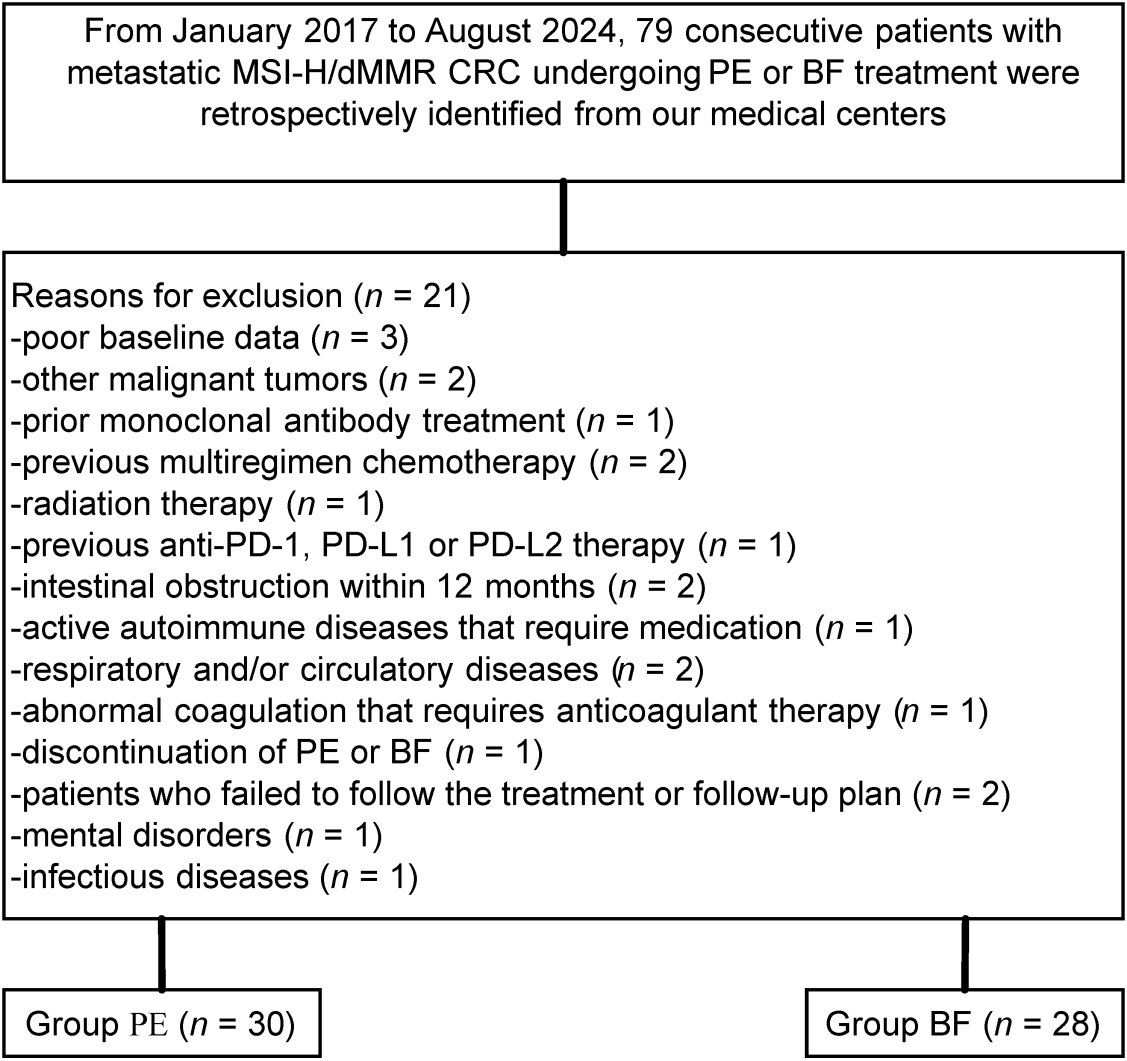
Flow diagram demonstrating the methods used to identify objects to evaluate the efficacy and safety of pembrolizumab versus bevacizumab plus modified FOLFOX6 in patients with metastatic, microsatellite instability–high/mismatch repair–deficient colorectal cancer.

Among the 79 individuals diagnosed with metastatic MSI-H/dMMR CRC included in this study, 21 were excluded based on predetermined criteria, resulting in a final sample size of 58 patients (PE cohort: n=30; BF cohort: n=28), as illustrated in **Figure 1**. The demographic and baseline characteristics of these individuals, for whom complete baseline data were accessible, are presented in **Table 1**. Overall, comparable demographic variables were observed between the cohorts, independent of potential comorbidities.

**Table 1:** Patient characteristics at baseline between two cohorts.

Variable	PE (n = 30)	BF (n = 28)	p-value
Age, years			
Median (range)	63.0 (34-82)	63.5 (32-84)	0.164 *^a^*
Sex, no. (%)			0.971 *^b^*
Female	13 (43.3)	12 (42.9)	
Male	17 (56.7)	16 (57.1)	
BMI, kg/m^2^			
Median (range)	23.3 (15.3-34.7)	23.9 (15.6-35.3)	0.182 *^a^*
ECOG PS, no. (%)			0.461 *^b^*
0**^#^**	9 (30.0)	11 (39.3)	
1**^##^**	21 (70.0)	17 (60.7)	
Primary tumor location, no. (%)			0.751 *^b^*
Right side (caecum to transverse colon)	18 (60.0)	16 (57.1)	
Left side (splenic flexure to rectum)	7 (23.3)	6 (21.4)	
Other side	5 (16.7)	6 (21.4)	
Modified FOLFOX6 cycles (range)	8 (6-10)	8 (6-11)	0.127 *^a^*
PD-L1 expression level (CPS values) ^$^, no. (%)			0.605 *^b^*
≥ 1	15 (50.0)	16 (57.1)	
20-50	10 (33.3)	8 (28.6)	
>50	5 (16.7)	4 (14.3)	
Specimens obtained from, no. (%)			0.886 *^b^*
Primary tumor	22 (73.3)	21 (75.0)	
Metastatic tumor	8 (26.7)	7 (25.0)	
Time since diagnosis, month (s)			
Median (range)	3 (1-6)	4 (1-7)	0.181 *^a^*
Duration of treatment, month (s)			
Median (range)	14 (1-24)	15 (1-24)	0.871 *^a^*

a Analysed using independent samples t-test.

b Analysed using Mann-Whitney U test; 0**^#^**, Fully active, able to carry on all pre-disease performance without restriction; 1**^##^**, Restricted in physically strenuous activity but ambulatory and able to perform work of a light or sedentary nature; **^$^** patients with higher PD-L1 expression was associated with a better survival.

PE, pembrolizumab; BF, bevacizumab plus modified FOLFOX6; BMI, body mass index; PS ECOG PS, Eastern Collaborative Oncology Group performance status; PD-L1, programmed cell death ligand-1; CPS, combined positive score.

At baseline, the median age in the PE group was 63.0 years (range 34–82 years), while the median age in the BF group was 63.5 years (range 32–84 years). The ECOG PS distribution among the PE cohort was 30.0% at 0 and 70.0% at 1, compared to the BF cohort, which had 39.3% at 0 and 60.7% at 1 (p=0.461). The primary tumor sites predominantly localized to the right colon (from the caecum to the transverse colon), comprising 60.0% in the PE group and 57.1% in the BF group (p=0.751). PD-L1 expression levels, as indicated by CPS values, showed that in the PE group, 50.0% had a CPS ≥ 1, 33.3% were within the 20-50 range, and 16.7% exceeded 50, whereas the BF group had 57.1% with CPS ≥ 1, 28.6% in the 20-50 range, and 14.3% exceeding 50 (p=0.605).

### Efficacy

The median follow-up duration for the study was 18.0 months (range 1.0–24.0 months). The tumor responses observed between the two cohorts are summarized in **Table 2**. In the PE cohort, 40.0% (95% CI, 34.3-42.6) of individuals achieved an objective response, which included 6.6% with complete responses and 33.3% with partial responses, while 16.7% demonstrated stable disease according to investigator assessment; 40.0% presented with progressive disease, and tumor response was unclear in 3.3% of cases. In contrast, the BF cohort exhibited an objective response rate of 17.8% (95% CI, 12.5-20.6), with 3.6% achieving complete responses and 10.7% partial responses, alongside 25.0% reaching stable disease; 53.6% had progressive disease, and 7.1% had unclear responses. Statistically significant differences were identified in the objective response rates between the cohorts, with 12 individuals (40.0%) in the PE group versus 4 individuals (17.8%) in the BF group (p=0.030). Notably, partial responses were more prevalent in the PE group compared to the BF group.

**Table 2:** Tumor response between two cohorts.

	PE (n = 30)	BF (n=28)	p*-*value*
ORR**^#^**, no. (%)	12 (40.0)	4 (17.8)	0.030
95% CI	34.3-42.6	12.5-20.6	
Overall response, no. (%)			0.024
CR	2 (6.6)	1 (3.6)	
PR	10 (33.3)	3 (10.7)	
SD	5 (16.7)	7 (25.0)	
PD	12 (40.0)	15 (53.6)	
Unclear	1 (3.3)	2 (7.1)	

Tumor response was evaluated by mRECIST per independent imaging review.

**^#^** The proportion of confirmed CR or PR per independent imaging review; *Analysed using Mann-Whitney U test.

PE, pembrolizumab; BF, bevacizumab plus modified FOLFOX6; mRECIST, modified Response Evaluation Criteria in Solid Tumors; ORR, objective response rate; CI, confidence interval; CR, complete response; PR, partial response; SD, stable disease; PD, progressive disease.

**Figure 2:**
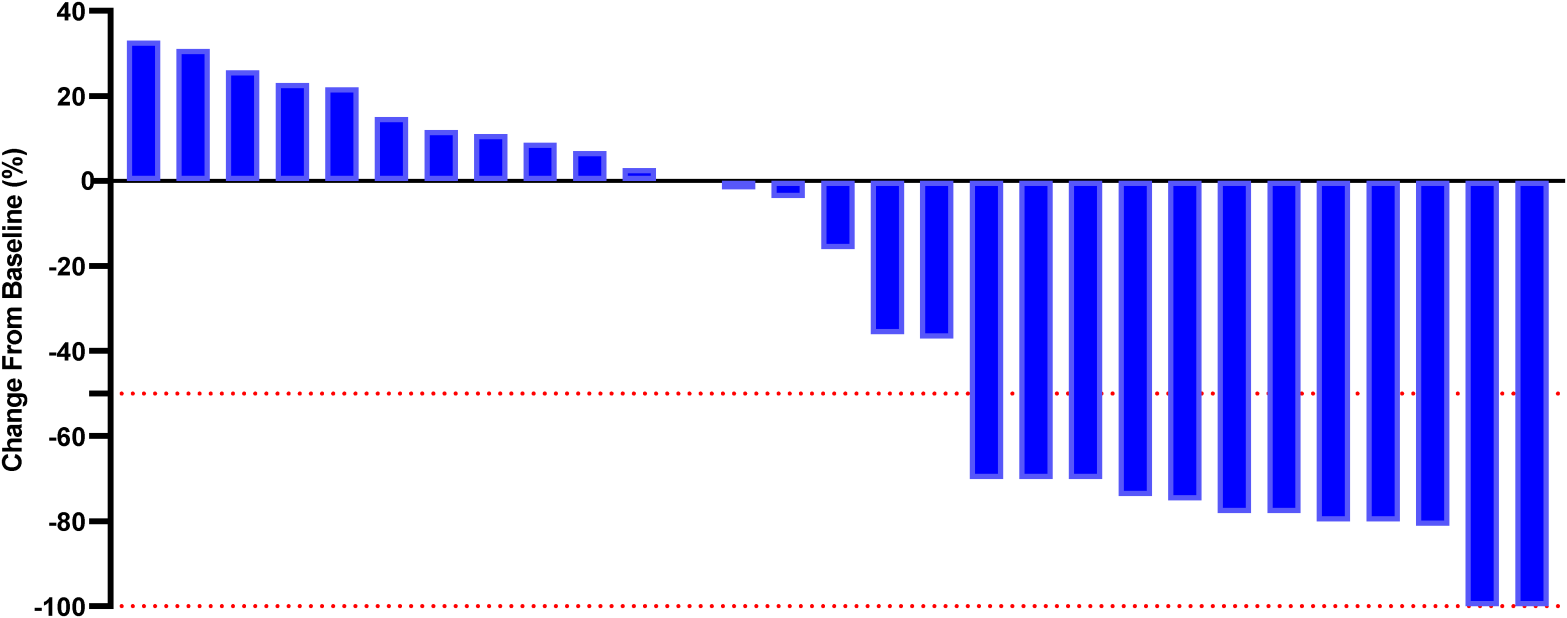
Percentage change from baseline in sums of diameters of target lesions by mRECIST in patients with metastatic MSI-H/dMMR CRC who experienced PE therapy (n = 29).

**Figure 3:**
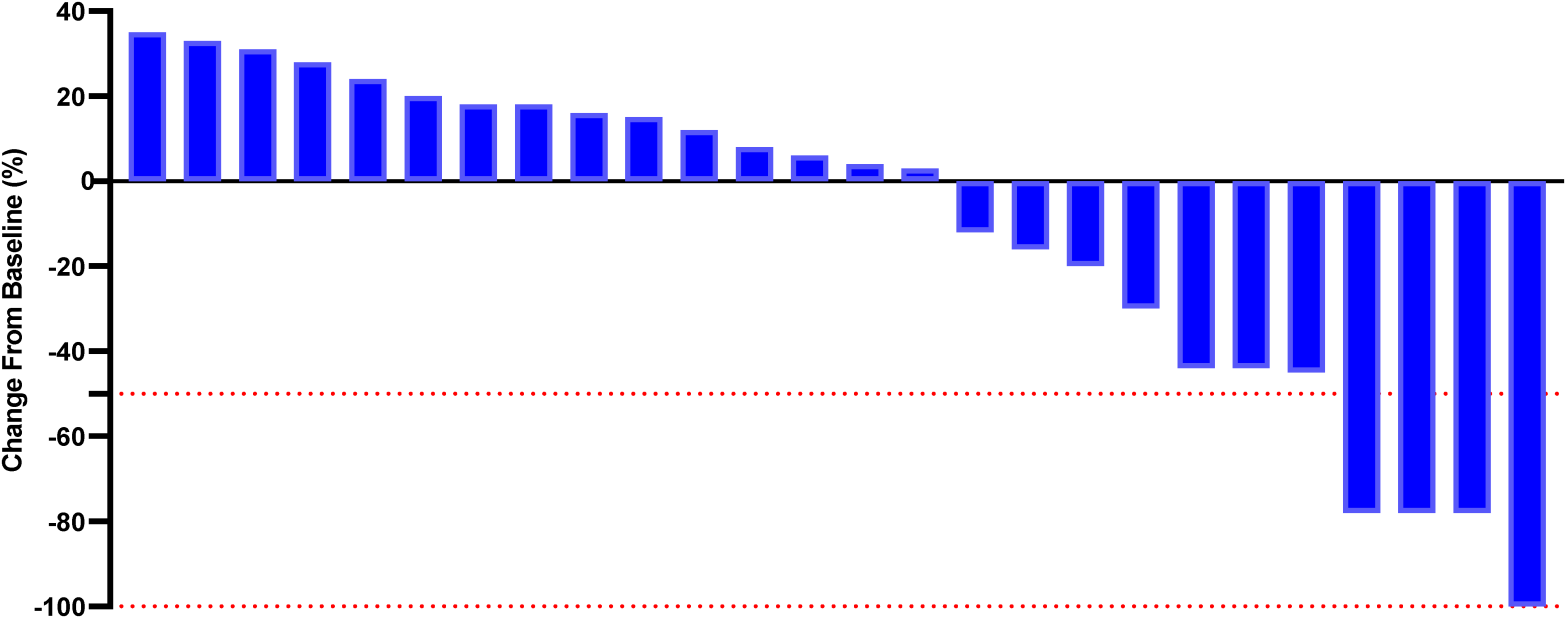
Percentage change from baseline in sums of diameters of target lesions by mRECIST in patients with metastatic MSI-H/dMMR CRC who experienced BF therapy (n = 26).

**Figure 4:**
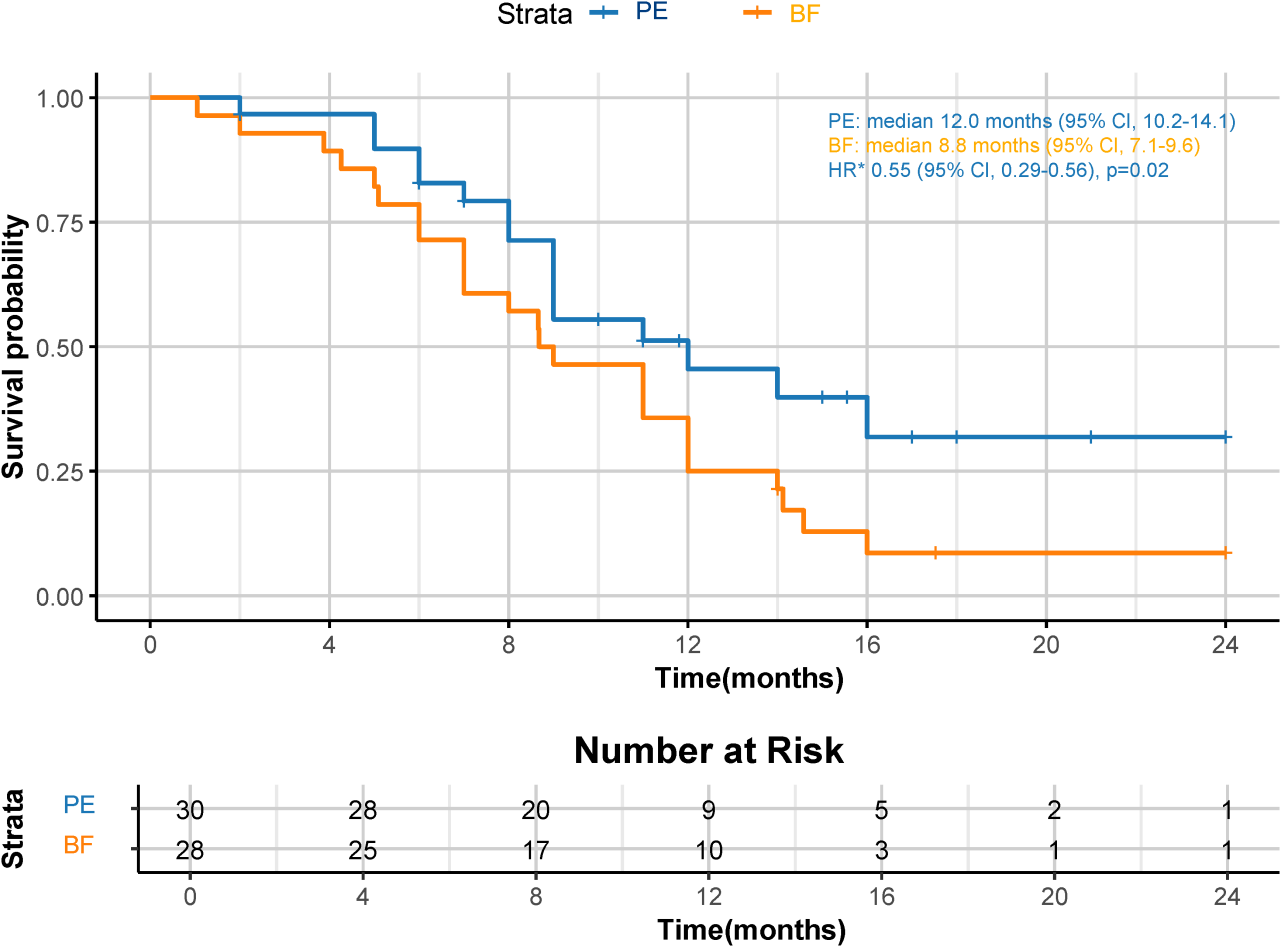
Kaplan-Meier curves for OS. The median OS was 12.0 months (95% CI, 10.2-14.1) for PE and 8.8 months (95% CI, 7.1-9.6) for BF (HR 0.55, 95% CI 0.29-0.56; p=0.02). *HR was calculated using a Cox proportional hazards model, with the age, sex, BMI, ECOG PS, primary tumor location, and PD-L1 expression level used as covariates and therapy as time-dependent factor. OS, overall survival; CPS, combined positive score; CI, confidence interval; PE, pembrolizumab; BF, bevacizumab plus modified FOLFOX6; HR, hazard ratio; BMI, body mass index; ECOG PS, Eastern Collaborative Oncology Group performance status; PD-L1, programmed cell death ligand-1.

**Figure 5:**
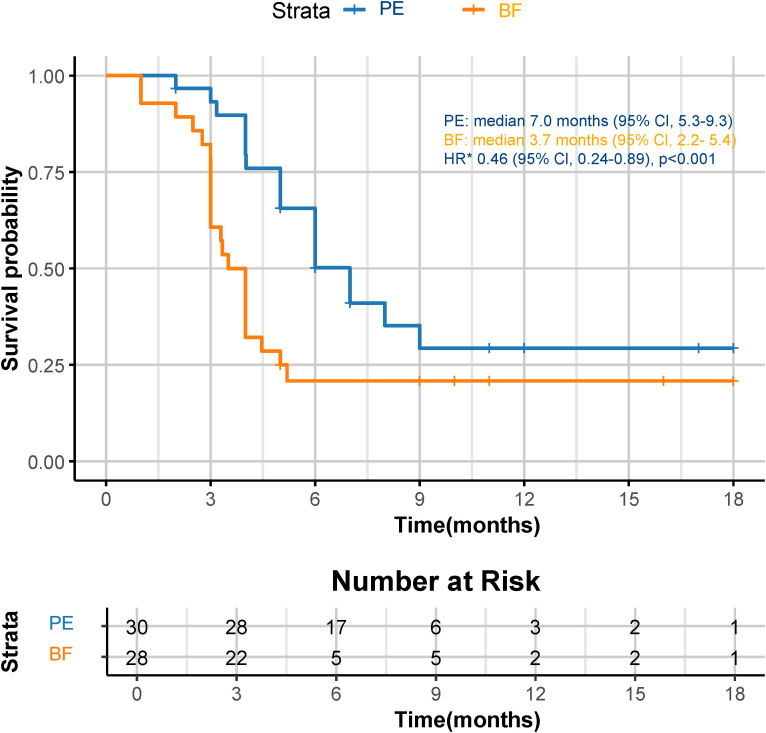
Kaplan-Meier curves for PFS. The median PFS was 7.0 months (95% CI, 5.3-9.3) for PE and 3.7 months (95% CI, 2.2-5.4) for BF (HR 0.46, 95% CI 0.24-0.89; p<0.001). *HR was calculated using a Cox proportional hazards model, with the age, sex, BMI, ECOG PS, primary tumor location, and PD-L1 expression level used as covariates and therapy as time-dependent factor. OS, overall survival; CPS, combined positive score; CI, confidence interval; PE, pembrolizumab; BF, bevacizumab plus modified FOLFOX6; HR, hazard ratio; BMI, body mass index; ECOG PS, Eastern Collaborative Oncology Group performance status; PD-L1, programmed cell death ligand-1.

Although a significant distinction in tumor size reduction was not observed (58.6% [17 of 29] in the PE cohort versus 42.3% [11 of 26] in the BF cohort; p=0.231), as depicted in **Figures 2** and **3**, the median overall survival (OS) was significantly greater in the PE-treated cohort at 12.0 months (95% CI, 10.2-14.1) compared to 8.8 months (95% CI, 7.1-9.6) in the BF-treated cohort (HR 0.55, 95% CI 0.29-0.56; p=0.02), as illustrated in **Figure 4**. Furthermore, the median progression-free survival (PFS) was extended in the PE cohort to 7.0 months (95% CI, 5.3-9.3) versus 3.7 months (95% CI, 2.2-5.4) in the BF cohort (HR 0.46, 95% CI 0.24-0.89; p<0.001) (**Figure 5**).

**Figure 6:**
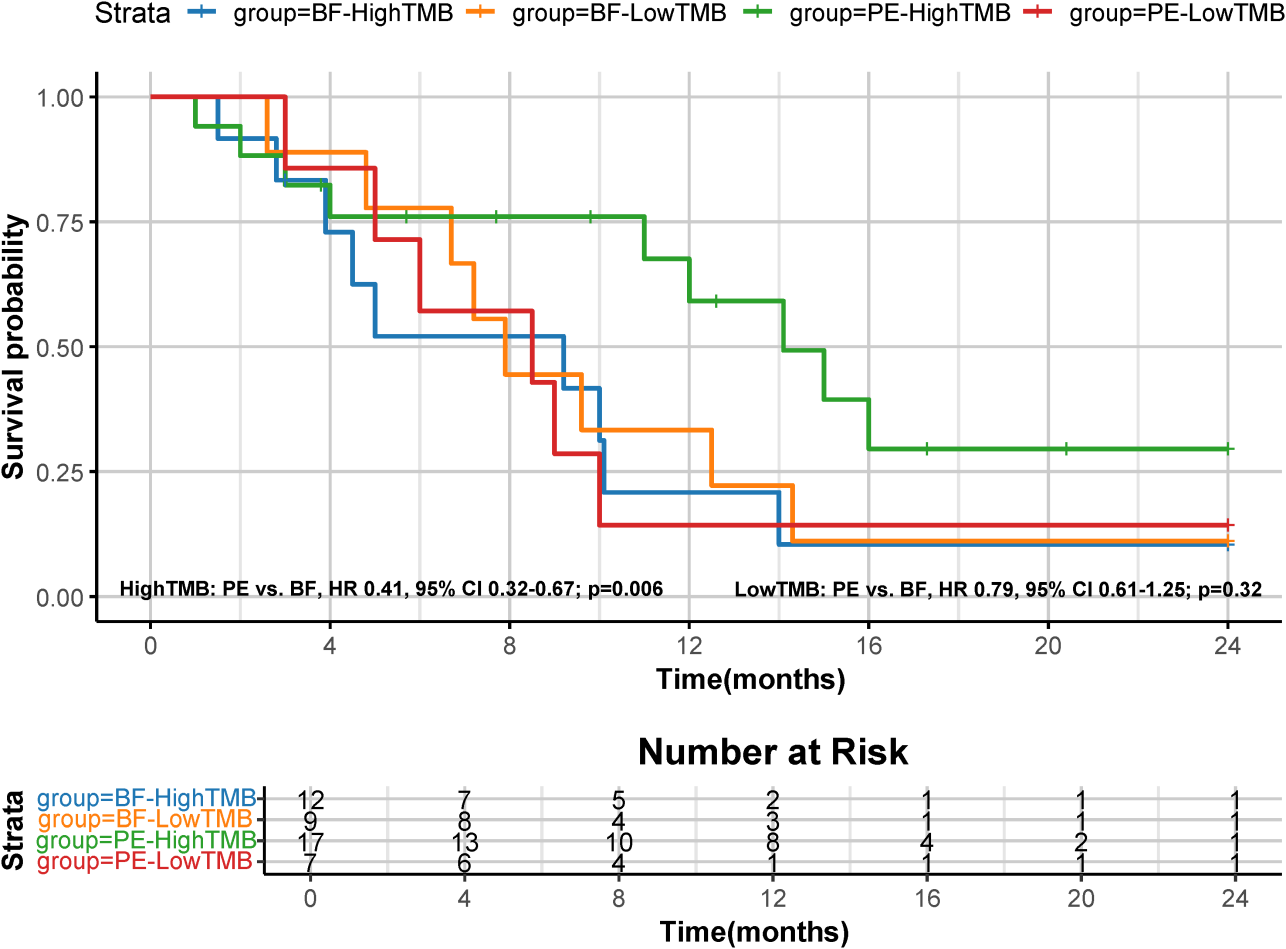
Kaplan-Meier curves for OS based on tumor mutation burden (TMB). The median OS was 14.1 months (95% CI, 12.3-16.8) for PE-high TMB patients and 9.2 months (95% CI, 7.4-10.1) for BF-high TMB patients (HR 0.41, 95% CI 0.32-0.67; p=0.006); The median OS was 8.5 months (95% CI, 6.5-9.8) for PE-low TMB patients and 7.9 months (95% CI, 6.4-9.7) for BF-low TMB patients (HR 0.79, 95% CI 0.61-1.25; p=0.32).

**Figure 7:**
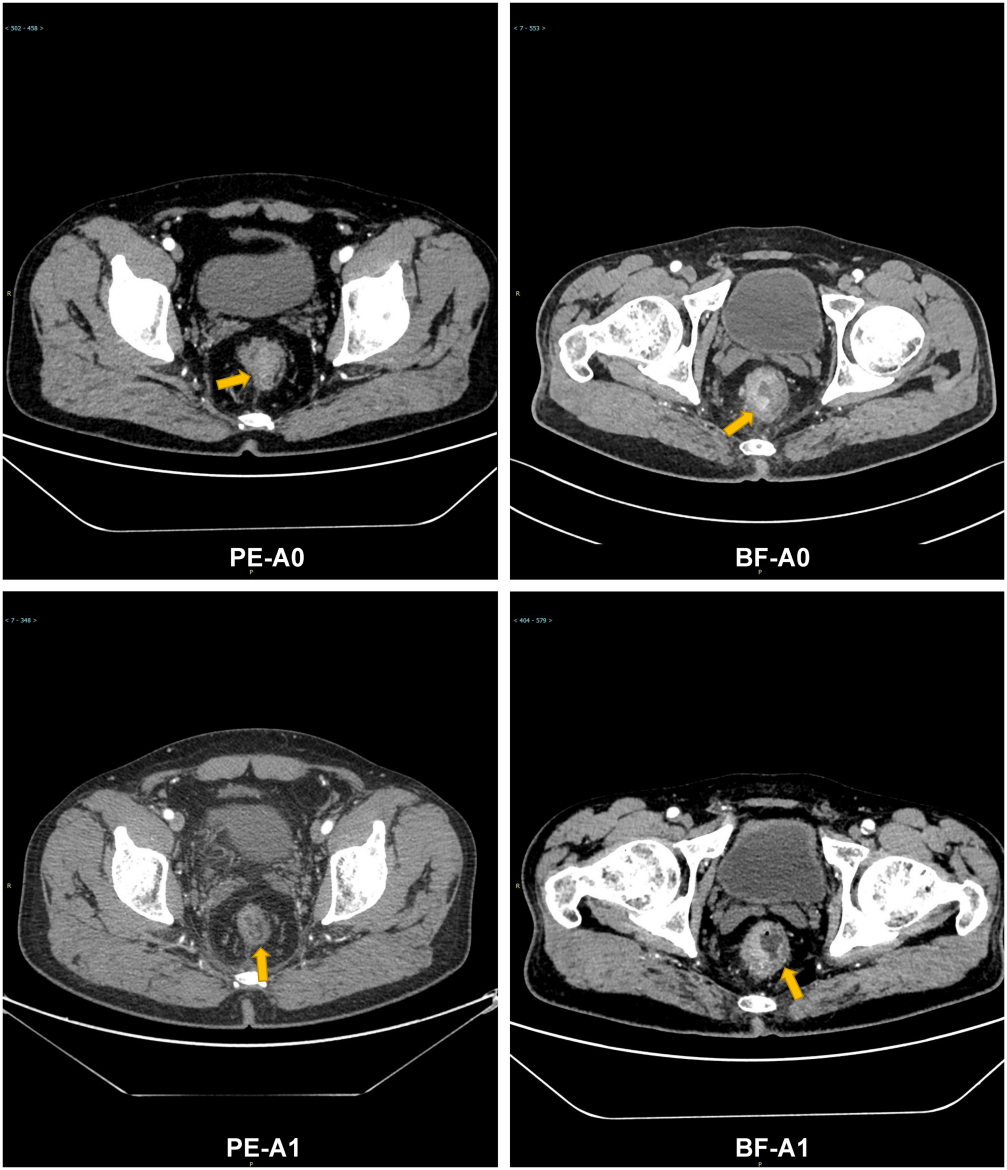
Comparative radiographic response of PE vs. BF in high TMB patients: tumor volume and Hounsfield unit calue changes at 6 months. Contrast-enhanced CT: PE-A0 (pre-treatment), PE-A1 (6-month post-PE); BF-A0 (pre-treatment), BF-A1 (6-month post-BF).

A *post hoc* analysis was performed using available NGS data for 45 patients (PE cohort, n=24: High TMB, n=17, and Low TMB, n=7; BF cohort, n=21: High TMB, n=12, and Low TMB, n=9). High TMB patients in the PE cohort showed superior median OS (14.1 months [95% CI: 12.3–16.8] vs. 9.2 months [95% CI: 7.4–10.1] in high TMB BF patients; HR=0.41, 95% CI 0.32-0.67; p=0.006), as shown in **Figure 6**. Low TMB patients exhibited no significant OS benefit between cohorts (PE: 8.5 months vs. BF: 7.9 months; HR=0.79, 95% CI 0.61-1.25; p=0.32). These results suggest that pembrolizumab’s survival advantage may be more pronounced in high TMB subgroups. **Figure 7** illustrates superior efficacy of PE therapy in high TMB patients compared to BF, with significant tumor shrinkage (e.g., mean Δvolume: −65% vs. −22%) and hounsfield unit reduction (ΔHU: −25 vs. −12) at 6-month follow-up, corroborating the survival advantage observed in **Figure 6**.

### Safety

**Table 3** outlines the incidence of grade ≥ 3 drug-related AEs. The safety profiles of the PE and BF cohorts were comparable, with no statistically significant intergroup differences noted in the rates of grade ≥ 3 drug-related AEs consistent with the monitored toxicity profiles. The adverse events observed aligned with the established safety profiles of both treatments. Hypertension was reported in one individual (3.3%) in the PE group, occurring within the first 12 months, while three individuals (10.7%) in the BF group experienced hypertension within the first 6 months. A dose reduction due to grade ≥ 3 drug-related AEs occurred in 4 individuals (2 individuals [6.7%] in the PE group and 2 individuals [7.1%] in the BF group; p=0.943).

**Table 3:** Treatment-related ≥ grade 3 AEs in patients who experienced PE or BF.

AEs, no. (%)	PE (n=30)	BE (n=28)	p-value*
Hypertension	1 (3.3)	3 (10.7)	0.272
Arthralgia	2 (3.3)	1 (3.5)	0.598
Nausea	1 (3.3)	2 (7.1)	0.516
Asthenia	2 (6.6)	2 (7.1)	0.943
Fatigue	1 (3.3)	1 (3.5)	0.961
Decreased appetite	0 (0.0)	1 (3.5)	0.301
Pruritus	2 (6.6)	1 (3.5)	0.598
Nausea	0 (0.0)	1 (3.2)	0.516
Diarrhea	0 (0.0)	1 (3.5)	0.301
Rash	1 (3.3)	1 (3.5)	0.961
Hypothyroidism	2 (6.6)	1 (3.5)	0.598
Hyperthyroidism	2 (6.6)	0 (0.0)	0.168
Pancreatitis	1 (3.3)	0 (0.0)	0.334
Pneumonitis	1 (3.3)	1 (3.5)	0.961
Myositis	1 (3.3)	0 (0.0)	0.334
Colitis	1 (3.3)	1 (3.5)	0.961

*Analyzed using Mann-Whitney U test. AEs, adverse events; PE, pembrolizumab; BF, bevacizumab plus modified FOLFOX6.

## Discussion

This study comprehensively evaluated the clinical outcomes of pembrolizumab versus bevacizumab in combination with modified FOLFOX6 in patients with metastatic MSI-H/dMMR CRC, with a median follow-up period of 18 months. To our knowledge, this retrospective analysis may represent the largest study conducted to date on the efficacy of PD-1 blocking antibodies versus anti-VEGF therapy specifically in Asian individuals with metastatic MSI-H/dMMR CRC. Our findings indicate that pembrolizumab therapy is a viable treatment option for this patient population. However, as a retrospective study, our results establish associations rather than causality. Caution is warranted in interpreting the observed superiority of pembrolizumab, and future prospective randomized trials are needed to validate these findings.

The results of this study corroborate findings from previous analogous studies (2, 22, 23), which explored the effectiveness of PD-1 inhibitors in patients with advanced MSI-H/dMMR CRC. For example, a multicenter study (24) involving pembrolizumab combined with chemotherapy demonstrated a median PFS of 8.8 months among patients with metastatic CRC, although the median OS was not reached. Similarly, a phase II trial (17) assessing the antitumor activity of nivolumab reported that 31.1% of patients experienced an objective response, with 68.9% achieving disease control for 12 weeks or longer. In that trial, the 9-month and 12-month PFS rates were 54% and 50%, respectively, alongside OS rates of 78% and 73%. Notably, the median follow-up in our study (18 months) may underestimate long-term survival benefits, particularly for pembrolizumab, given the potential “tail effect” of immunotherapy. Extended longitudinal follow-up is critical to assess the durability of responses.

The superior efficacy of pembrolizumab in MSI-H/dMMR CRC is rooted in the unique immunobiology of these tumors (6). MSI-H/dMMR tumors exhibit deficient DNA mismatch repair, leading to hypermutated genomes and the accumulation of neoantigens (1, 2, 7). These neoantigens serve as immunogenic targets, promoting cytotoxic T-cell infiltration and activation (12, 24–26). Pembrolizumab, a PD-1 inhibitor, disrupts the PD-1/PD-L1 axis, which is frequently hijacked by tumors to evade immune surveillance (27). By blocking this checkpoint, pembrolizumab restores T-cell-mediated tumor killing, as evidenced by increased CD8+ T-cell density and clonal expansion in responders (8, 26).

Recent studies (28–30) highlight the dynamic interplay between MSI-H status and the tumor microenvironment. For instance, MSI-H tumors are characterized by a “hot” immune phenotype, marked by elevated interferon-γ signaling, upregulated antigen-presenting machinery, and enhanced PD-L1 expression (31, 32). These features create a permissive environment for PD-1 inhibitors, whereas microsatellite stable (MSS) tumors often exhibit an immunosuppressive TME dominated by regulatory T cells (Tregs) and myeloid-derived suppressor cells (MDSCs) (19, 33). However, even within MSI-H/dMMR CRC, heterogeneity exists. High tumor mutational burden (TMB ≥10 mutations/Mb) correlates with increased neoantigen load and improved pembrolizumab response (34, 35). Conversely, low TMB or high tumor burden may foster an immunosuppressive niche via hypoxia-driven upregulation of VEGF and other angiogenic factors, which inhibit dendritic cell maturation and T-cell trafficking (25, 27). This aligns with our findings that bevacizumab—an anti-VEGF agent—combined with chemotherapy may transiently benefit high tumor burden patients by normalizing aberrant vasculature and reducing immunosuppressive cytokines (12, 36).

While pembrolizumab enhances adaptive immunity, bevacizumab primarily targets the TME’s vascular infrastructure (37). Bevacizumab inhibits VEGF-A, reducing angiogenesis and vascular permeability, which may transiently improve drug delivery and alleviate tumor hypoxia (4, 13, 26). However, prolonged VEGF blockade can paradoxically induce immunosuppression by promoting Treg infiltration and impairing dendritic cell function (2, 11, 34). In contrast, pembrolizumab sustains T-cell activation and memory responses, potentially explaining its prolonged survival benefits despite lower initial response rates in high tumor burden settings (12, 18).

Emerging evidence (13, 17) suggests synergistic potential when combining PD-1 inhibitors with anti-angiogenic agents. Preclinical models demonstrate that VEGF inhibition can reprogram the TME by reducing Tregs and enhancing CD8+ T-cell infiltration (14, 19, 38), while PD-1 blockade prevents T-cell exhaustion (12). Clinical studies (13, 39, 40) exploring this combination in MSI-H/dMMR CRC are ongoing and may address the limitations of monotherapy in high-risk subgroups.

As with all observational studies, several limitations of the current investigation should be acknowledged. First, the retrospective design inherently introduces challenges, including unmeasured confounding variables (e.g., tumor microenvironment heterogeneity, treatment preferences) and selection bias, which may affect causal interpretations. Second, our cohort exclusively comprised Asian patients, limiting generalizability to other ethnic populations where genetic, environmental, or immunological differences (e.g., HLA diversity, gut microbiota) may influence outcomes. Third, the lack of systematic quantification of tumor burden—such as metastatic lesion count, total tumor volume, or ctDNA dynamics—represents a critical gap, as high tumor burden is strongly associated with immunotherapy resistance and may have biased efficacy comparisons between regimens (34, 35). Fourth, TMB was retrospectively assessed in only 77.6% of patients (45/58), and its predictive utility requires prospective validation. Additionally, other biomarkers—including driver mutations (e.g., KRAS/BRAF), immune cell infiltration, and tumor microenvironment features—were not evaluated but may further elucidate response heterogeneity. Finally, the median follow-up of 18 months may underestimate long-term survival benefits, particularly for pembrolizumab, given the delayed responses characteristic of immunotherapy.

## Conclusion

This study demonstrates superior survival outcomes with pembrolizumab versus bevacizumab-based chemotherapy in metastatic MSI-H/dMMR CRC, alongside manageable toxicity. TMB stratification revealed pronounced benefits in TMB-high (≥10 mut/Mb) patients, while low-TMB/high-burden subgroups may require combination therapies targeting immune and angiogenic pathways. The efficacy of PD-1 inhibition aligns with MSI-H tumors’ neoantigen-driven immunogenicity, enabling T-cell activation through checkpoint blockade. These findings support pembrolizumab as a standard therapy for this population. Future randomized trials should prioritize multidimensional biomarkers (TMB, ctDNA, tumor burden) to refine patient selection and validate long-term outcomes across diverse cohorts.

## Data Availability

The original contributions presented in the study are included in the article/supplementary material. Further inquiries can be directed to the corresponding authors.
